# Association between the rs2288947 polymorphism of the lncRNA *TINCR* gene and the risk of recurrent miscarriage in a Southern Chinese population

**DOI:** 10.1002/jcla.22919

**Published:** 2019-05-23

**Authors:** Wendong Huang, Huazhong Zhou, Lei Pi, Yufen Xu, LanYan Fu, Yanfang Yang, Di Che, Xiaoqiong Gu

**Affiliations:** ^1^ Department of Pharmacy Maoming People's Hospital Maoming China; ^2^ Department of Clinical Biological Resource Bank, Guangzhou Institute of Pediatrics, Guangzhou Women and Children's Medical Center Guangzhou Medical University Guangzhou China; ^3^ Department of Prenatal Diagnosis Maoming People's Hospital Maoming China; ^4^ Department of Blood Transfusion, Guangzhou Women and Children's Medical Center Guangzhou Medical University Guangzhou China; ^5^ Department of Clinical Laboratory, Guangzhou Women and Children's Medical Center Guangzhou Medical University Guangzhou China

**Keywords:** genetic susceptibility, lncRNA TINCR, recurrent miscarriage

## Abstract

Studies have shown that many genes that regulate cell migration are associated with susceptibility to recurrent miscarriage. Terminal differentiation‐induced non‐coding RNA (TINCR) regulates the migration and invasion of a variety of tumor cells and is associated with susceptibility to various diseases. However, whether the lncRNA TINCR polymorphism is associated with susceptibility to recurrent miscarriage is unclear. Therefore, we investigated the relationship between the rs2288947 A > G polymorphism of the lncRNA TINCR and susceptibility to recurrent abortion. We recruited 248 recurrent spontaneous abortion patients and 392 healthy control subjects from the Southern Chinese population and used the TaqMan method for genotyping. There was no evidence that this polymorphism is associated with recurrent miscarriage (AG vs AA: adjusted OR = 0.904, 95% CI = 0.647‐1.264, *P* = 0.5552; GG and AA: adjusted OR = 0.871, 95% CI = 0.475‐1.597, *P* = 0.6542; dominant model: AG/GG vs AA: adjusted OR = 0.898, 95% CI = 0.653‐1.236, *P* = 0.5101; and recessive model: GG vs AA/AG: adjusted OR = 0.910, 95% CI = 0.505‐1.639, *P* = 0.7527). The stratified analysis also showed no significant associations. This study suggests that the rs2288947 A > G polymorphism of the lncRNA TINCR may not be associated with recurrent miscarriage in a Southern Chinese population. A larger multicenter study is needed to confirm our conclusions.

## 1 INTRODUCTION

Recurrent miscarriage is a multifactorial disease, and the cause is unknown in approximately half of the patients with recurrent spontaneous abortion.^1^ The occurrence of recurrent miscarriage is associated with genetics, uterine abnormalities, thrombotic tendency, hormone or metabolic disorders, infection, autoimmunity, age, and lifestyle problems.^1^ Studies have found that susceptibility genes have an important relationship to the pathogenesis of recurrent spontaneous abortion.^2^ In recent years, with the increase in research on recurrent abortion, an increasing number of susceptibility genes, such as MMP2, MMP9, CTLA4, FOXP3, and THBD, have been found to be associated with susceptibility to recurrent miscarriage.^3^, ^4^, ^5^, ^6^ In addition, studies have confirmed that some genes that regulate cell migration are associated with susceptibility to recurrent miscarriage^7^, ^8^ Studies have also found that long non‐coding RNA (lncRNA) polymorphisms are also associated with recurrent miscarriage. For example, in the early stage, we studied MALAT1, which regulates cell migration and is associated with susceptibility to recurrent miscarriage. Therefore, exploring the relationship between lncRNA and the function of regulating cell migration and recurrent abortion provides an important reference for understanding the causes of recurrent miscarriage.

Terminal differentiation‐induced non‐coding RNA (TINCR) is a lncRNA that is located on human chromosome 19 and produces a 3.7‐kb transcript.^9^, ^10^ Studies have shown that TINCR is involved in epidermal differentiation and somatic tissue differentiation.^10^, ^11^ Multiple studies have revealed that TINCR is involved in tumorigenesis and aberrant expression in multiple human cancers.^12^, ^13^, ^14^ Moreover, TINCR has been shown to regulate proliferation, migration, and invasion of multiple tumor cells.^15^, ^16^, ^17^, ^18^ Recently, studies have found that genetic variation in TINCR was associated with a variety of diseases, such as the SNP rs2288947, which contributed to the susceptibility and progression of colorectal cancer and susceptibility to gastric cancer.^19^, ^20^ Furthermore, studies have found that some genes that regulate cell proliferation, invasion, and migration are associated with susceptibility to miscarriage; for example, H19 and p53 gene polymorphisms.^8^, ^21^ These studies suggest that TINCR gene polymorphisms may be associated with recurrent miscarriage. However, there have been no previous studies on the association between TINCR polymorphism rs2288947 A > G and recurrent miscarriage; therefore, we conducted this study.

## 2 MATERIALS AND METHODS

### 2.1 Ethics statement

The study was approved by the Ethics Review Committee of the Guangzhou Women and Children's Medical Center. Written informed consent was obtained from each participant.

### 2.2 Subjects

From June 2017 to July 2018, 248 women diagnosed with recurrent miscarriage and 392 healthy controls were collected from the Department of Gynecology, Guangzhou Women and Children's Medical Center. Recurrent miscarriage was defined as two or more spontaneous abortions with unknown etiology. The control women had at least two normal pregnancies and no history of miscarriage. Recurrent miscarriage patients and control women had no autoimmune, liver or kidney dysfunction; history of endocrine or metabolic disorders; arterial or venous thrombosis; abnormal uterine anomalies; hypertension; or embryo chromosomal abnormalities.

### 2.3 Genotyping and DNA extraction

We isolated total genomic DNA from peripheral blood leukocytes using the TIANamp Blood DNA Kit (TianGen Biotech Co., Ltd.). Genotyping of SNPs rs2288947 was performed in 384‐well plates by the TaqMan real‐time polymerase chain reaction protocol on an ABI Q6 (Thermo Fisher Scientific). In addition, approximately 10% of samples were randomly selected for sequencing for quality control purposes and validation of genotyping results. The results were 100% concordant.

### 2.4 Statistical analysis

Hardy‐Weinberg equilibrium (HWE) of the control subjects was calculated by the goodness‐of‐fit chi‐square test. Demographic and genotypic differences between cases and controls of recurrent spontaneous abortion were compared by the chi‐square test. Unconditional univariate and multivariate logistic regression analyses were performed. Age‐adjusted odds ratios (OR) and 95% confidence intervals (CI) were used to assess the strength of the association between this genetic polymorphism and the susceptibility to recurrent miscarriage. Stratified analysis of age and number of abortions was performed. A *P*‐value of less than 0.05 was considered statistically significant, and all statistical tests were bilateral and calculated using SAS software (version 9.1; SAS Institute).

## 3 RESULTS

### 3.1 Population characteristics

The demographic characteristics of recurrent spontaneous abortion and control groups are shown in Table 1. There was no statistically significant difference in age (31.00 ± 4.83 vs 31.44 ± 4.39 years, *P* = 0.7225) between the case and the control group. In recurrent miscarriage patients, approximately 68.15% of recurrent miscarriage patients experienced two or three spontaneous abortions, and more than 31.85% experienced four or more spontaneous abortions.

**Table 1:** Frequency distribution of selected characteristics in recurrent miscarriage and controls

Variables	Cases (n = 248)		Controls (n = 392)		*P* a
	No.	%	No.	%	
Age range, year	20‐44		22‐44		
Mean ± SD	31.00 ± 4.83		31.44 ± 4.39		0.7225
**<**35	187	75.4	288	73.47	
35‐40	52	20.97	92	23.47	
>40	9	3.63	12	3.06	
No. of abortions/%					
2‐3	169	68.15			
≥4	79	31.85			

a Two‐sided chi‐square test for distributions between recurrent miscarriage patients and controls.

### 3.2 Association between selected polymorphism and recurrent miscarriage susceptibility

The genotype frequencies of the TINCR gene rs2288947 polymorphism in patients with recurrent miscarriage and the control group are listed in Table 2. Genotype analysis of the TINCR gene rs2288947 showed no significant difference in HWE in the control group (*P* = 9731). We did not observe any significant association between the TINCR gene rs2288947 polymorphism and susceptibility to recurrent miscarriage (AG vs AA: adjusted OR = 0.904, 95% CI = 0.647‐1.264, *P* = 0.5552; GG and AA: adjusted OR = 0.871, 95% CI = 0.475‐1.597, *P* = 0.6542; dominant model: AG/GG vs AA: adjusted OR = 0.898, 95% CI = 0.653‐1.236, *P* = 0.5101; and recessive model: GG vs AA/AG: adjusted OR = 0.910, 95% CI = 0.505‐1.639, *P* = 0.7527).

**Table 2:** Genotype and allele frequencies of TINCR in RM patients and controls

Genotype/ allele	RM (N = 248）	Controls (N = 392)	*P*‐value^a^	OR (95% CI)	*P*‐value	Adjusted OR (95% CI)	*P*‐value^b^
TINCR/rs2288947 A > G (HWE = 0.9731)							
AA	132 (53.23)	198 (50.51)		1.00	/	1.00	/
AG	97 (39.11)	161 (41.07)	/	0.904 (0.647‐1.263)	0.5532	0.904 (0.647‐1.264)	0.5552
GG	19 (7.66)	33 (8.42)	/	0.864 (0.471‐1.583)	0.6353	0.871 (0.475‐1.597)	0.6542
Additive			0.7908	0.918 (0.715‐1.179)	0.5031	0.920 (0.716‐1.182)	0.5157
Dominant	116 (46.77)	194 (49.49)	0.503	0.897 (0.652‐1.233)	0.5031	0.898 (0.653‐1.236)	0.5101
Recessive	229 (92.34)	359 (91.58)	0.7318	0.903 (0.501‐1.625)	0.7328	0.910 (0.505‐1.639)	0.7527

Abbreviation: RM: Recurrent miscarriage.

a Chi‐square test for genotype distributions between recurrent miscarriage patients and controls.

b Adjusted for age.

### 3.3 Stratified analysis of selected polymorphisms and recurrent miscarriage susceptibility

As shown in Table 3, we further evaluated the role of the TINCR gene rs2288947 polymorphism in cases and controls by stratified analysis. Participants were stratified according to age and the number of spontaneous abortions. The results showed that there was no significant correlation between the TINCR gene rs2288947 polymorphism and the risk of recurrent miscarriage in different age groups or the number of spontaneous abortions.

**Table 3:** Stratification analysis for associations between TINCR polymorphism and recurrent miscarriage risk in a south Chinese population

Variable	rs2288947 (cases/controls)		*P*	OR (95% CI)	*P*	Adjust OR (95% CI)	*P* a
	AG/GG	AA					
Age							
<35	88/143	99/145	0.5805	0.901 (0.623‐1.303)	0.5806	/	/
35‐40	22/44	30/48	0.5227	0.800 (0.403‐1.588)	0.5235	/	/
>40	6/7	3/5	0.6963	1.429 (0.236‐8.637)	0.6976	/	/
No. of abortions/%							
2‐3	90/198	79/194	0.5506	0.896 (0.624‐1.286)	0.5508	0.898(0.625‐1.291)	0.563
≥4	42/198	37/194	0.6667	0.899 (0.554‐1.459)	0.6669	0.894(0.551‐1.453)	0.652

a Adjusted for age.

## 4 DISCUSSION

In the current case‐control study with 248 patients (with recurrent miscarriage) and 396 healthy controls from a Southern Chinese population, we did not observe a significant relationship between the TINCR gene rs2288947 A > G polymorphism and the recurrent spontaneous abortion susceptibility of women in Southern China. In addition, in the stratified analysis, we did not observe any significant associations in different age groups with recurrent spontaneous abortion or in the number of spontaneous abortions. To the best of our knowledge, this is the first study to investigate the relationship between the TINCR gene rs2288947 A > G polymorphism and the susceptibility to recurrent miscarriage.

Although the specific functions of most lncRNAs are still unknown, recent studies have shown that lncRNAs play an important role in the development and progression of abortion.^22^, ^23^ Feng et al^24^ clarified that many lncRNAs are abnormally expressed in patients with recurrent spontaneous abortion and that lncRNA can be used as a biomarker for predicting recurrent spontaneous abortion. A study by Zhang et al^25^ suggested that lncRNAs HOTAIR may be a potential therapeutic target for recurrent miscarriage. Recently, a study by Che et al found that the genetic susceptibility gene lncRNA CCAT2, which regulates cell invasion and migration, is associated with RSA susceptibility. Very recently, studies have confirmed that the lncRNA TINCR gene, which regulates cell invasion and migration, is associated with susceptibility to various diseases. A study by Ma et al^20^ found that SNPs of the lncRNA TINCR were significantly associated with decreased gastric cancer susceptibility by decreasing its gene expression levels. Moreover, a study by Zheng et al^19^ found that lncRNA TINCR polymorphisms were associated with the progression of colorectal cancer, and SNP rs2288947 may be a biomarker with the occurrence and progression of colorectal cancer. In our case‐control study with 248 patients with recurrent miscarriage and 392 healthy controls from the Southern Chinese population, we did not observe a significant relationship between the TINCR gene rs2288947 A > G polymorphism and the recurrent abortion susceptibility of women in Southern China. In the stratified analysis, the lncRNA TINCR rs2288947 A > G polymorphism was not significantly associated with the number of abortions or age groups. Our current study suggests that the lncRNA TINCR rs2288947 A > G polymorphism may not be suitable as a biomarker for the diagnosis or prognosis of recurrent abortion. However, a larger sample size is needed to confirm these results.

Although this is the first study to investigate the association between the TINCR gene rs2288947 A > G polymorphism and the risk of recurrent abortion in China, our study has several limitations that need to be addressed. First, due to the retrospective nature of the original research design, we have no detailed information about other factors, such as lifestyle habits, smoking, drinking, and other risk factors for miscarriage. Second, we only studied the rs2288947 A > G polymorphism, and other potential SNPs for the TINCR gene were not included in this study. In the next study, we will study the relationship between polymorphisms at other loci (such as rs8105637 G > A and rs8113645 G > A) and susceptibility to recurrent abortion. Third, we conducted a case‐control study to investigate the relationship between the TINCR gene rs2288947 A > G polymorphism and susceptibility to recurrent miscarriage. We did not investigate the expression level or potential mechanism of action of TINCR. Fourth, this study included only 248 patients with recurrent spontaneous abortion. The number of cases was small and may have limited statistical power. In our next study, we will attempt to verify the association in a larger sample size at multiple centers. Moreover, we encourage replication studies with larger sample sizes at other centers to validate the association.

In summary, we conducted a case‐control study with 248 patients with recurrent spontaneous abortion and 392 healthy controls from a Southern Chinese population. We did not observe a significant association between the TINCR gene rs2288947 A > G polymorphism and recurrent miscarriage and other parameters, such as age and the number of spontaneous abortions. However, larger‐scale, multicenter studies that have larger sample sizes, different races, and more polymorphisms are needed to confirm our findings.

## CONFLICT OF INTEREST

The authors report no conflicts of interest.

## AUTHOR CONTRIBUTIONS

All authors contributed significantly to this work. DC and WD H devised the research plan. The data were analyzed by YF Y and QHL. DC wrote the manuscript, and YQT, ZLL, and HZZ were responsible for performing the experiments. LP and LYF designed the experimental methods, and QFL modified and polished the manuscript. All authors support the publication of the manuscript.

## Data Availability

Data sharing is not applicable to this article because no datasets were generated or analyzed during the current study. Please contact the author with any data requests.
